# Green Extraction of Volatile Terpenes from *Artemisia annua* L.

**DOI:** 10.3390/molecules30071638

**Published:** 2025-04-07

**Authors:** Marta Mandić, Ivona Ivančić, Matija Cvetnić, Claudio Ferrante, Giustino Orlando, Sanda Vladimir-Knežević

**Affiliations:** 1 Faculty of Pharmacy, University of Mostar, Matice Hrvatske BB, 88000 Mostar, Bosnia and Herzegovina; marta.mandic@farf.sum.ba (M.M.); ivona.ivancic@farf.sum.ba (I.I.); 2 Faculty of Chemical Engineering and Technology, University of Zagreb, Trg Marka Marulića 19, 10000 Zagreb, Croatia; mcvetnic@fkit.unizg.hr; 3 Department of Pharmacy, D’Annunzio University of Chieti-Pescara, Via dei Vestini, 31, 66013 Chieti, Italy; claudio.ferrante@unich.it (C.F.); giustino.orlando@unich.it (G.O.); 4 Faculty of Pharmacy and Biochemistry, University of Zagreb, Ante Kovačića 1, 10000 Zagreb, Croatia

**Keywords:** *Artemisia annua*, sweet wormwood, essential oil, terpenes, green extraction, hydrodistillation, steam distillation, supercritical CO_2_ extraction, artemisia ketone, arteannuin B

## Abstract

In the present study, the extraction of volatile terpenes from *A. annua* with supercritical CO_2_ (sc-CO_2_) was optimized by a full factorial design procedure and compared with conventional distillation. The influence of pressure (100–220 bar) and temperature (40–60 °C) on sc-CO_2_ extraction was investigated to obtain extracts rich in the desired components while maintaining a high yield. Extraction yields (m/m) varied from 0.62% (130 bar/40 °C) to 1.92% (100 bar/60 °C). Monoterpenes were the most abundant constituents of the sc-CO_2_ extracts, among which artemisia ketone (16.93–48.49%), camphor (3.29–18.44%) and 1,8-cineole (4.77–11.89%) dominated. Arteannuin B (3.98–10.03%) and β-selinene (1.05–7.42%) were the major sesquiterpenes. Differences were found between the terpene profiles of the sc-CO_2_ extracts and the essential oils obtained by conventional hydrodistillation and steam distillation, as well as between the distilled essential oils. Our results demonstrate the optimal conditions for the rapid and effective supercritical extraction of certain monoterpenes and sesquiterpenes from *A. annua*, which have promising antimicrobial, antioxidant, antiviral, anti-inflammatory and antitumor properties.

## 1. Introduction

*Artemisia* L. is one of the largest and most widespread genera in the Asteraceae family. It comprises more than 500 taxa that are widely distributed in the temperate regions of the northern hemisphere, with only a few representatives in the southern hemisphere. *Artemisia* species mainly colonize dry and semi-arid habitats, from sea level to high altitudes, but they are able to thrive and survive in almost all habitat types. Central Asia is the center of their origin and their main center of diversification, while the Mediterranean region and Northwest America are two secondary speciation areas [1,2]. They are herbaceous annual, biennial and perennial plants or compact shrubs known for their characteristic bitter taste and pungent odor due to the presence of sesquiterpene lactones and other terpenoids [3,4]. *Artemisia* species have a rich history in traditional medicine around the world and provide remedies for a variety of ailments [5]. In addition to their promising traditional use, modern scientific research continues to investigate the efficacy and safety of *Artemisia* species in various therapeutic contexts. These medicinal plants gained much research attention in 2015, when the Nobel Prize in Physiology and Medicine was awarded for the discovery of artemisinin, a sesquiterpene lactone isolated from the Chinese herb *A. annua* L. (sweet wormwood), which is widely used as an antimalarial agent. The discovery of artemisinin has dramatically changed the landscape in the fight against malaria and has led to a paradigm shift in the development of antimalarial drugs [6,7].

*A. annua* has been used in traditional Asian medicine, especially in Chinese and Hindu medicine, for more than 2000 years to treat fever in various diseases [8]. *A. annua* has significant health benefits, particularly in the fight against malaria, but it also shows promise in other therapeutic areas, including the treatment of viral and bacterial diseases and even cancer. Namely, extensive scientific research has shown that *A. annua* has numerous other biological activities, such as action against other diseases caused by protozoa; antibacterial, antifungal and antiviral activities; and immunosuppressive, anti-inflammatory, anticancer, analgesic, nephroprotective, antioxidant and anti-obesity effects [9,10,11,12]. Due to its high biological value, the plant is increasingly used as an ingredient in cosmetic products for the protection and care of skin, hair and nails [8,13].

The most important bioactive components of *A. annua* are mainly specific sesquiterpene lactones, essential oil, flavonoids, phenolic acids and coumarins. The geographical location and habitat in which the plant grows, the time of harvest, the drying conditions and the extraction method have a major influence on the phytochemical profile of the plant and thus on its biological properties [10]. Previous studies have shown that the essential oil, which consists mainly of monoterpenes and sesquiterpenes, contributes significantly to the biological effects and health benefits of *A. annua*, but the content and composition of the essential oil are subject to considerable variability [8]. Due to its geographical origin, the essential oil of *A. annua* showed large differences in the contents of the main components artemisia ketone (3,3,6-trimethyl-1,5-heptadien-4-one), 1,8-cineole, camphor and germacrene D [14,15]. Furthermore, an intraspecific chemical diversity within the species was observed based on the essential oil profile [16]. The essential oil composition is certainly influenced by the extraction methods used. Hydrodistillation and solvent extraction are the traditional methods used to obtain the essential oil of *A. annua*. The extraction method is one of the most important elements in determining the quality of essential oils, as their chemical signature, intrinsic properties and bioactivity can be affected. For example, high temperatures and the presence of water during hydrodistillation can lead to degradation and chemical alteration of the essential oils, while the essential oils obtained by extraction with organic solvents usually contain toxic solvent residues [17,18]. Supercritical fluid extraction (SFE) has proven to be an innovative method for isolating essential oils, as it allows extracts to be obtained without solvent contamination and uses mild processing conditions that avoid the degradation of heat-sensitive compounds.

This extraction method is advantageous due to its “green” properties and milder temperature conditions, but it also enables selectivity and efficiency due to the properties of a supercritical fluid. The SFE utilizes the improved diffusivity and low viscosity of the supercritical fluid, which facilitates the efficient extraction of analytes from complex matrices. Supercritical carbon dioxide (sc-CO_2_) is most commonly used as it is non-flammable, low toxic, inexpensive and easy to remove from the extract. A CO_2_ recycling system in SFE is associated with lower costs and lower environmental impact, which is in line with the principles of green chemistry [19,20]. The extraction yield and the composition of the extracts proved to be useful criteria for comparing SFE with conventional extraction methods. For example, the essential oil was isolated from *Eucalyptus loxophleba* leaves by the parallel application of SFE, Soxhlet extraction and hydrodistillation. The highest yield was achieved by Soxhlet extraction with ethanol, and the lowest was achieved by hydrodistillation. In Soxhlet extraction, the type of solvent had a greater influence on the yield than the extraction time, while in hydrodistillation, the yield increased with the extraction time. The addition of ethanol as a co-solvent improved the efficiency of the SFE. The hydrodistilled essential oil contained only volatile components, while the other two samples contained both volatile and higher molecular weight compounds. Accordingly, the content of 1,8-cineole was highest in the distilled essential oil and lowest in the Soxhlet extract [21]. A comparison of the essential oils extracted from different *Salvia* species using sc-CO_2_ and simultaneous distillation/extraction showed that the yield of the supercritical extracts was significantly lower [22]. The yield and chemical profile of *Thymus munbyanus* essential oils obtained by SFE and hydrodistillation were also compared. The yield of the supercritical extract was higher than that of the hydrodistilled essential oil but with a significantly lower number of volatile terpenes and more terpenes with a higher molecular mass [23]. Supercritical CO_2_ permeates solid matrices and selectively dissolves the target compounds without the need for large amounts of organic solvents. However, co-solvents can be added as CO_2_ does not have the necessary polarity to extract polar substances. The SFE process is optimized by adjusting the properties of the supercritical fluid. It has been found that pressure, temperature, co-solvent concentration, sample size, flow rate and extraction time influence the efficiency of SFE [20].

In line with all the above, our study primarily aimed to optimize the environmentally friendly, mild and rapid process of supercritical CO_2_ extraction of volatile terpenes from leaves and flowering tops of *A. annua* using a full factorial design approach. We focused our research on obtaining extracts that are rich in the desired components and, at the same time, have a high yield. A second objective was to compare the terpene profile of the sc-CO_2_ extracts with the essential oils obtained by hydrodistillation and steam distillation.

## 2. Results and Discussion

### 2.1. Content and Composition of Essential Oil of Artemisia annua Obtained by Hydrodistillation and Steam Distillation

The essential oil was isolated from the flowering tops and leaves of *A. annua* by hydrodistillation and steam distillation for three hours. Hydrodistillation yielded 3.24% (V/m) of the essential oil, while steam distillation yielded 0.50% (V/m). It is difficult to compare the yields obtained with previous studies, as many factors, such as the parts of the plant collected, the time of harvest, the drying method, the geographical region or the method and duration of the distillation process, play an important role. Previous studies have shown that the essential oil yield of *A. annua* obtained by hydrodistillation during the flowering period from August to November can vary between 0.37% and 2.25% [24,25,26,27,28,29,30,31,32]. It was also found that hydrodistillation of the flowering tops and leaves gave a higher yield of essential oil (0.99%) than the whole aerial parts (0.66%) of *A. annua* [29]. Steam distillation is much less commonly used in essential oil research than hydrodistillation, which is also true for *A. annua.* It has been reported that the yield of essential oil of *A. annua* from various regions of Korea obtained by steam distillation ranged from 0.04% to 1.09% [16].

**Figure 1:**
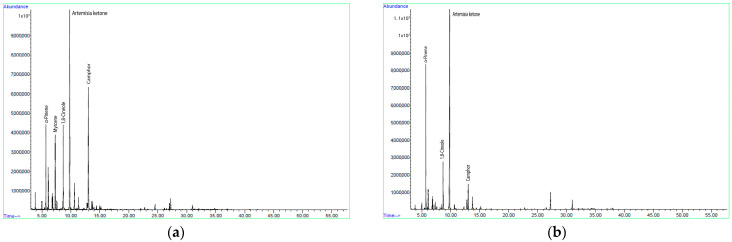
Gas chromatograms of the essential oil obtained by hydrodistillation (**a**) and steam distillation (**b**) from dried leaves and flowering tops of *Artemisia annua* L.

According to the results of GC-MS analysis, 34 and 26 different compounds were identified in the essential oils from leaves and flowering tops obtained by hydrodistillation and steam distillation, which accounted for 97.55% and 95.04% of the total oil content, respectively (Table 1). Both essential oil samples were characterized by a very high content of monoterpenes (91.62% and 88.13%, respectively), while sesquiterpenes were only weakly represented. The monoterpenes were predominantly present in oxidized form, while hydrocarbons were the most abundant of the sesquiterpenes. Despite the significant differences in content observed, artemisia ketone was the major constituent of both the essential oil obtained by hydrodistillation (29.20%) and the essential oil obtained by steam distillation (42.04%) (Figure 1). The hydrodistilled essential oil contained significant amounts of camphor (17.67%), 1–8 cineole (9.88%), myrcene (7.73%) and α-pinene (7.64%), while the steam-distilled oil contained more α-pinene (17.78%) and less of the other components mentioned (1.11–7.47%). Among the sesquiterpenes, β-selinene (1.74–3.70%) and caryophyllene oxide (0.90–2.26%) were the most abundant.

The hydrodistilled essential oil profile is consistent with the results of previous studies due to the high content of oxidized forms of monoterpenes, with the unusual artemisia ketone and the most common 1,8-cineole and camphor being the main representatives. The essential oil profile obtained was most similar to the Italian samples of the flowering tops and aerial parts, which were characterized by high levels of artemisia ketone (17.3–24.3%), camphor (17.0–22.6%) and 1,8-cineole (16.0–19.0%) [24,26,29,33]. In some other essential oils isolated mainly from aerial parts, such as those from Serbia [25,34], Bosnia and Herzegovina [35], Morocco [27] and China [36], artemisia ketone was the main component, followed by α-pinene, camphor or β-caryophyllene. In contrast, the Iranian [37], Brazilian (leaves) [38] and Romanian [28] essential oil samples did not contain artemisia ketone as the major component but 1,8-cineole and camphor. Thus, previous studies have shown that the essential oil composition of *A. annua* varies greatly depending on the geographical origin. A review of the literature data revealed several chemotypes depending on the proportions of the following components: camphor, 1,8-cineole, artemisia ketone and germacrene D [15]. A hierarchical cluster analysis of the essential oils of *A. annua* from Tajikistan revealed the existence of three chemotypes: camphor/1,8-cineole, camphor and artemisia ketone [14], while a study of Serbian samples revealed five different chemotypes in terms of the content of artemisia ketone, α-pinene, 1,8-cineole, germacrene D and β-caryophyllene [25]. A multivariate analysis of 103 samples of steam-distilled oils isolated from *A. annua* growing in different Korean regions divided them into three main groups: artemisia ketone, 1,8-cineole and β-selinene [16]. The essential oil obtained by steam distillation in our study corresponds well to the artemisia ketone cluster as it contains more than 41% artemisia ketone. Regarding the influence of the extraction method on the essential oil composition, our results also showed that direct steam distillation at atmospheric pressure favors the extraction of artemisia ketone and α-pinene, while hydrodistillation gives a lower percentage of artemisia ketone and is more suitable for camphor extraction.

In agreement with previous studies, our results also indicate the complexity of the chemical composition of *A. annua* essential oil, which certainly determines its biological activity, such as antimicrobial and antioxidant effects. Previous studies showed that essential oils rich in artemisia ketone (12.5–43.2%) had strong antibacterial and antifungal activity against *Escherichia coli*, *Enterococcus faecalis*, *Candida albicans*, *Malassezia sympodialis* and *Fusarium oxysporum* [27,33,39]. Essential oils with a high camphor content (7.1–43.5%) also had strong antimicrobial activity against various bacteria and fungi, such as *Escherichia coli*, *Enterococcus hirae*, *Staphylococcus aureus*, *Candida albicans*, *Saccharomyces cerevisiae*, *Trichophyton rubrum* and *Epidermophyton floccosum* [40,41,42]. The essential oil of *A. annua* has been found to have antioxidant properties, especially when the sesquiterpene fraction is particularly abundant alongside artemisia ketone or camphor as the main constituents [27,42]. Radulović et al. [25] reported that artemisia ketone has stronger antioxidant properties compared to camphor, 1,8-cineole and α-pinene. It also showed the highest antimicrobial activity against tested Gram-positive and Gram-negative bacterial strains and fungal strains, with *Staphylococcus aureus* being the most sensitive. The author also suggested that the use of *A. annua* essential oil does not pose a health risk, as the essential oil showed no negative effects on kidney and liver function and morphology in rats.

### 2.2. Content and Composition of sc-CO_2_ Extracts of Artemisia annua Depending on the Applied Pressure and Temperature—Full Factorial Design Approach

The optimization of sc-CO_2_ extraction of volatile terpenes from the flowering tops and leaves of *A. annua* was performed by a full factorial design with two factors: pressure at five levels and temperature at three levels. The pressure range was between 100 and 220 bar, while the temperature varied between 40 and 60 °C. The extraction time of half an hour and the CO_2_ flow rate of 50 g/min remained constant during the extraction process. In view of the results obtained, the response surface method was used with the Design Expert 8 software. Table 2 shows the yield of the sc-CO_2_ extracts as a function of the applied pressure and temperature. The extraction yield varied from 0.62% (sequence 8) to 1.92% (sequence 3). The yield of 1.92% was obtained at the lowest pressure of 100 bar and the highest temperature of 60 °C.

**Figure 2:**
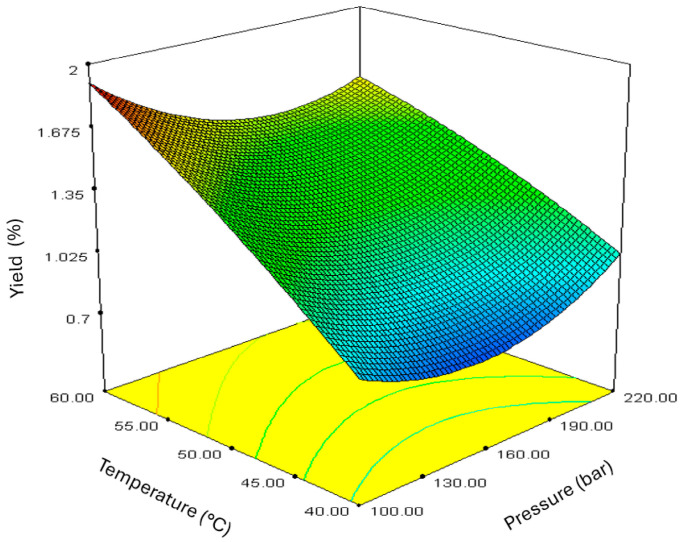
Three-dimensional plot of the sc-CO_2_ extraction yield of *Artemisia annua* L. as a function of pressure and temperature.

The influence of the pressure and temperature was investigated through a variance analysis (ANOVA) with 95% confidence (*p* ≤ 0.05). According to the ANOVA results presented in Supplementary Materials (Table S1), the regression model for the extraction yield was significant (*p* = 0.0125) with a satisfactory coefficient of determination (R^2^). The temperature was the most statistically significant parameter for the observed response (*p* = 0.0007), indicating its strong influence on the yield of sc-CO_2_ extracts of *A. annua*. In contrast, statistical analysis showed no significant effect of applied pressure on the extraction yield. The model was used to create a response surface that shows the extraction yield as a function of temperature and pressure (Figure 2). It can be seen that at constant pressure, an increase in temperature from 40 to 60 °C leads to a linear increase in extraction yield. The response surface shows the lowest yield at low temperatures, and with increasing temperature, the yield increases visibly. In contrast, there is no such positive trend when the pressure increases at a constant temperature. The surface shows that the yield is highest at extreme pressure, while the medium pressures promote the lowest yield.

Previous research on the extraction of *A. annua* with supercritical fluids was mainly aimed at finding the most efficient conditions for the extraction of artemisinin and its derivatives in comparison or combination with solvent extraction [43,44,45,46]. However, reports on the optimization of extraction conditions for essential oil from *A. annua* are very rare. Moreover, the extraction conditions used vary considerably, which further complicates the comparison of the results. Li et al. reported that efficient extraction of essential oil from *A. annua* can be performed for two hours at a pressure of 250 bar, a temperature of 50 °C and a CO_2_ flow rate of 25 g/min. The highest positive effect on extraction was attributed to extraction time and pressure. The authors also pointed out that the combination of supercritical fluid extraction and molecular distillation is a good means of obtaining high-quality essential oils [17]. The influence of pressure (100, 200 and 300 bar) and temperature (40 and 60 °C) on the yield of sc-CO_2_ extracts was also investigated by Vidović et al. During a three-hour extraction, they achieved the highest yield at 300 bar and 40 °C. The authors linked the increase in yield to an increase in pressure at a constant temperature [47]. In contrast, our results showed a stronger influence of temperature on the extraction yield compared to the influence of pressure. When considering this difference, it should be borne in mind that our extraction time was much shorter than in the two previous studies, and the applied pressure was a maximum of 220 bar. The reasons for a lower or higher extraction yield may also be related to the two basic properties of CO_2_ in the supercritical state, namely its low viscosity and its high diffusivity. With increasing pressure, the density also increases, which can contribute to a better solvation capacity and, thus, to a better extraction yield. An increase in temperature at constant pressure leads to a decrease in CO_2_ density but also to an increase in the vapor pressure of the components [19,48,49]. Therefore, the influence of temperature on the extraction yield depends on the relation between the density of the supercritical fluid and the vapor pressure of the components, i.e., which parameter has the predominant influence on the extraction. In our case, the vapor pressure of the components was obviously dominant, which can explain the much greater influence of temperature on the extraction yield compared to pressure.

**Figure 3:**
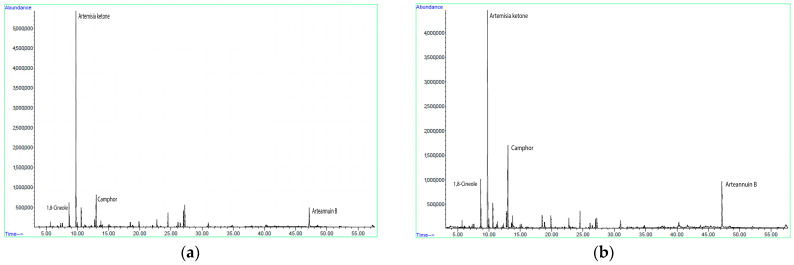
Gas chromatograms of the sc-CO_2_ extracts of *Artemisia annua* L. with the high content of artemisia ketone (**a**) and arteannuin B (**b**), obtained at 100 bar and 40 °C (sequence 15) and 220 bar and 60 °C (sequence 1), respectively.

The chemical composition of fifteen sc-CO_2_ extracts was analyzed by gas chromatography coupled with mass spectrometry (GC-MS). Thirty-two compounds were identified, accounting for 84.43–97.19% of the total crude oil (Table 3). The major volatile terpenes identified in the sc-CO_2_ extracts obtained were monoterpenes (59.43–80.16%), among which oxidized forms predominated (50.26–75.98%), such as artemisia ketone (16.93–48.49%), followed by camphor (3.29–18.44%), 1,8-cineole (4.77–11.89%), *trans*-pinocarveol (1.39–5.08%) and artemisia alcohol (2.25–4.10%) (Figure 3). The sesquiterpene fraction was less abundant (15.99–29.73%). In contrast to the monoterpenes, there were more hydrocarbons (9.95–18.59%) than oxidized forms (1.64–12.35%) among the sesquiterpenes. The most abundant sesquiterpenes were arteannuin B (3.98–10.03%), β-selinene (1.05–7.42%) and *trans*-β-caryophyllene (2.53–5.41%). Although the primary aim of sc-CO_2_ extraction of *A. annua* in previous studies was to separate artemisinin, some volatile compounds were also extracted so that we could use them for a rough comparison with the sc-CO_2_ extracts obtained in this work. Our results are in line with previous findings on the high content of camphor and 1,8-cineole [44,45], but our results are most comparable to those of Banožić et al. [50], where the most abundant compounds in the sc-CO_2_ extract were also arteannuin B, camphor and artemisia ketone. Other components consistent with our results were 1,8-cineole, *trans*-pinocarveol, artemisia alcohol, β-selinene and *trans*-β-caryophyllene, although artemisinin and artemisinic acid were not present in our extracts, probably due to the different extraction conditions.

In our study, the supercritical extracts contained fewer monoterpenes in number and total yield than the essential oil samples obtained by distillation, but the number and total yield of sesquiterpenes were significantly higher. In addition to an increased content of *trans*-β-caryophyllene, germacrene D and β-selinene, the extracts contained arteannuin B and arteannuic acid, which were not present in the hydrodistilled and steam-distilled essential oils of *A. annua*. The sc-CO_2_ extracts rich in sesquiterpenes, especially arteannuin B and β-selinene, in addition to their antimicrobial and antioxidant activity, indicate other aspects of the biological activity and potential therapeutic applications of *A. annua* extracts. Recent studies on arteannuin B have demonstrated its antitumor activity and low toxicity in vitro and in vivo [51]. Arteannuin B has been found to enhance the efficacy of cisplatin in non-small cell lung cancer by regulating connexin 43 and the mitogen-activated protein kinase signaling pathway [52]. It proved to be an inhibitor of the SARS-CoV-2 main protease (nonstructural protein 5) and caspase-8, the cysteine protease enzymes that are promising molecular targets for antiviral and anticancer agents [53]. Considerable antiviral effect of arteannuin B has been demonstrated in vitro, supporting the activity of *A. annua* against SARS-CoV-2 and other coronaviruses [12]. In a more recent study, this sesquiterpene lactone from *A. annua* was identified as a potent anti-inflammatory agent. It was found to attenuate the inflammatory response by suppressing the uncontrolled activation of the NF-κB signaling pathway [54]. In addition, previous research has also indicated the potential anti-inflammatory properties of β-selinene. *Callicarpa macrophylla* essential oil, which is rich in β-selinene (37.51–57.01%), showed anti-inflammatory, analgesic and antipyretic effects in the Swiss albino mice model compared to the standard drugs [55]. *Beta*-selinene was identified as an anti-inflammatory biomarker from essential oils of the Myrtaceae family by untargeted metabolomics [56]. The proportion of sesquiterpenes in the sc-CO_2_ extracts obtained in this study was significantly higher compared to the essential oils isolated by conventional distillation methods. Indeed, the presence of sesquiterpenes in sc-CO_2_ extracts extends the spectrum of biological effects of *A. annua* beyond the antimicrobial and antioxidant activity commonly associated with the monoterpenes in distilled essential oils. Our results suggest the potential of supercritical extracts for future research into anti-inflammatory, anticancer and antiviral effects.

**Figure 4:**
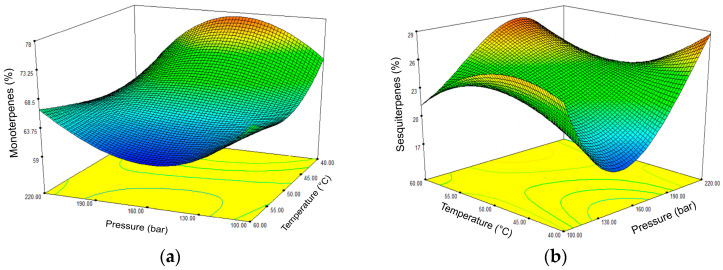
Three-dimensional plot of the content of monoterpenes (**a**) and sesquiterpenes (**b**) in sc-CO_2_ extracts of *Artemisia annua* L. as a function of pressure and temperature.

The regression model for the extraction of monoterpenes and sesquiterpenes proved to be significant, with a *p*-value of 0.0473 and 0.0433, respectively. The R^2^ values were up to 0.85. Temperature was statistically significant for both groups of compounds (*p* = 0.0019 and 0.0029), as were the relationships between temperature and pressure (Tables S2 and S3). Figure 4 shows the effects of temperature and pressure on the content of monoterpenes (a) and sesquiterpenes (b) in the obtained sc-CO_2_ extracts of *A. annua*. It can be seen that most of the monoterpenes are extracted at 40 °C and 160 bar or 50 °C and 190 bar. Moreover, increasing the temperature or pressure above these values has no positive effect on the extraction yield. For the extraction of sesquiterpenes, a less abundant fraction in sc-CO_2_ extracts, pressures of 160 and 190 bar are also suitable but with higher temperatures of 50 and 60 °C. A similar amount of sesquiterpenes can be obtained at a lower temperature of 40 °C, but then a higher pressure of 220 bar must be used.

**Figure 5:**
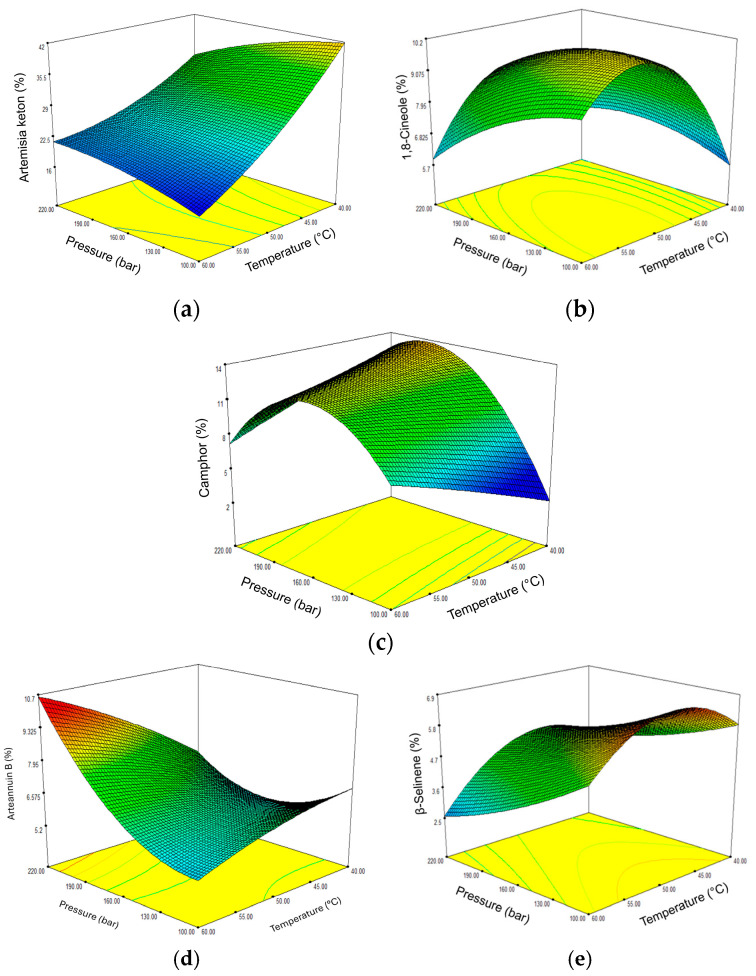
Three-dimensional plot of the content of the most abundant components in sc-CO_2_ extracts of *Artemisia annua* L. as a function of pressure and temperature. (**a**) Artemisia ketone; (**b**) 1,8-cineole; (**c**) camphor; (**d**) arteannuin B; (**e**) β-selinene.

The biological activity of a plant extract is determined by the content and composition of the active ingredients, which can be modulated by varying the extraction parameters. The most abundant terpenes were used for further statistical analysis, and the results of the proposed ANOVA models were summarized (Tables S4–S8). Based on the results obtained, regression models with significant variables (*p* = 0.0104–0.0480) were constructed for all target terpenes. Figure 5 shows the response surface plots for selected monoterpenes and sesquiterpenes as a function of temperature and pressure. 

In the case of artemisia ketone, the main component of all extracts, temperature proved to be the most important parameter (*p* = 0.0018), while the influence of pressure was not significant (Table S4). The response surface clearly shows that a low temperature favors a better extraction and that an increase in temperature leads to extracts with lower amounts of artemisia ketones (Figure 5a). The extracts richest in artemisia ketone were obtained at 40 °C and a pressure of 160 and 100 bar. Low temperatures most likely promote efficient extraction of artemisia ketone by maintaining high fluid density, minimizing thermal degradation and preventing volatilization losses. In contrast to the results for artemisia ketone, temperature was found to have no significant effect on camphor yield, but the most important parameters were pressure (*p* = 0.0155) and the relationship between pressure and temperature (*p* = 0.0198) (Table S6). The most efficient extraction of camphor occurs at 50 °C and pressures of 190 and 160 bar (Figure 5c). Accordingly, the best extraction of 1,8-cineole was also obtained at 50 °C, but at lower pressures of 100 and 130 bar (Figure 5b). Temperature was the only significant parameter (*p* = 0.0080) for 1,8-cineole extraction (Table S5).

In the sesquiterpene fraction of the sc-CO_2_ extract of *A. annua*, the sesquiterpene lactone arteannuin B and the sesquiterpene hydrocarbon β-selinene were predominant. The sc-CO_2_ extracts with the highest proportions of arteannuin B were obtained at the highest applied pressure and temperature. Figure 5d shows that its percentage increases linearly with increasing pressure from 160 to 220 bar and that the values increase with increasing temperature at the same pressures. The extraction of arteannuin B is most affected by pressure (*p* = 0.0133) and the relationship between pressure and temperature (*p* = 0.0264) (Table S7). Pressure and temperature were found to have a statistically strong effect on the extraction of β-selinene (Table S8). Extraction was most efficient at 50 °C and a low pressure of 100 to 160 bar, whereupon the content of β-selinene decreased. A similar trend was observed at both lower and higher temperatures (Figure 5e).

The response surface plots of the most abundant monoterpenes and sesquiterpenes in sc-CO_2_ extracts shown in Figure 4 indicate that they differ from each other, suggesting different effects of temperature and pressure on their extraction. Thus, pressure was found to be critical for the extraction of camphor, arteannuin B and β-selinene, while temperature was a significant variable for the yield of artemisia ketone, 1,8-cineole and β-selinene. The relationship between pressure and temperature was an additional significant variable for camphor and arteannuin B. The complex composition of such extracts and the synergistic-antagonistic effect of the ingredients certainly have a considerable influence on their biological activity. Our optimization therefore aimed to obtain the highest possible overall yields of the extracts with the highest possible contents of selected main constituents, which significantly determine the biological properties of the sc-CO_2_ extract under consideration. Table 4 provides the optimal extraction conditions for the selected targets, with the expected desirability function in the range of 0.473–0.899.

A temperature of 60 °C and a pressure of 100 bar proved to be optimal for the extraction of monoterpenes. If only the high content of artemisia ketone is required, the temperature must be lowered to 45 °C at the same pressure. However, to obtain extracts rich in all three most abundant monoterpenes (artemisia ketone, camphor and 1,8-cineole), the temperature and pressure must be increased slightly to 50 °C and 160 bar, respectively. The sesquiterpenes are also best extracted at 60 °C, but at a higher pressure (181 bar) than for the monoterpenes. For extracts rich in arteannuin B and β-selinene, an even higher pressure of 220 bar must be used. If a high proportion of arteannuin B is desired, a temperature of 60 °C must be maintained, while a slightly lower temperature of 53 °C is more suitable if, in addition to arteannuin B, a high proportion of β-selinene is also important. To ensure the quality, consistency and bioactive potency of the optimized sc-CO_2_ extracts of *A. annua*, it is important to select appropriate chemical markers among the main bioactive ingredients and to develop analytical methods for their fingerprint profiling and quality control.

## 3. Materials and Methods

### 3.1. Plant Material

The aerial parts of cultivated *Artemisia annua* L. were collected during the flowering period in September 2022 and 2023 in Bosnia and Herzegovina, in Ljubinje (42°57′04.4″ N 18°05′14.2″ E in Eastern Herzegovina). The plant was authenticated by the Department of Pharmacognosy, Faculty of Pharmacy and Biochemistry (University of Zagreb, Croatia), where the voucher specimen was also deposited. The collected plant material was air-dried in the shade at room temperature. The leaves and flowering tops were separated for examination.

### 3.2. Isolation of Essential Oil by Hydrodistillation and Steam Distillation

The essential oil of *A. annua* was isolated by hydrodistillation according to the European Pharmacopeia [57], while steam distillation was performed using a steam distillation apparatus (Šurlan–production of laboratory glassware, Medulin, Croatia). The dried plant material (20.0 g) was distilled for three hours in both distillation procedures. The yield (%, V/m) was calculated as the volume of essential oil (mL) per 100 g of plant material. After extraction, the collected essential oils were dried under anhydrous sodium sulfate and stored in a refrigerator at 4 °C.

### 3.3. Supercritical Carbon Dioxide (sc-CO_2_) Extraction

The extraction of the volatile terpene components from *A. annua* was carried out using an sc-CO_2_ extraction system (SFE Process, Tomblaine, France). A total of 10.0 g of ground plant material was placed in an extraction vessel with a volume of 100 mL. High-purity CO_2_ was used as solvent at a constant flow rate of 50 g/min. The extraction was carried out for 30 min at different temperatures (40–60 °C) and pressures (100–220 bar). The extracts obtained were collected in previously weighed glass tubes using a balance with an accuracy of ±0.0001 g and stored in a refrigerator at 4 °C. The extraction yield was expressed as mass percent (%, m/m).

A full factorial design was used to determine the optimal pressure and temperature to obtain the highest extraction yield and target ingredients. The commercial software Design-Expert^®^ (Ver. 8, Stat-Ease Inc., Minneapolis, MN, USA) was used to analyze the results. The quality of the fitted model was assessed using analysis of variance (ANOVA). The test for statistical differences was based on the total error with a confidence level of 95.0%.

### 3.4. GC-MS Analysis

The supercritical CO_2_ extracts and essential oils were analyzed using an Agilent 7890A gas chromatograph coupled to an Agilent 5975C electron ionization mass detector (Santa Clara, CA, USA). An aliquot of the sample (1 μL) dissolved in hexane (1:100) was injected into a split/splitless inlet at 240 °C, with a split ratio of 1:100. Helium was used as the carrier gas at a constant flow rate of 1.2 mL/min. The components were separated on a non-polar Agilent Technologies HP-5 MS capillary column (30 m × 0.25 mm, film thickness 0.25 μm). The following temperature program was used: isothermal at 45 °C for 2 min, increasing from 45 °C to 250 °C at a rate of 4 °C/min and holding isothermal at 250 °C for 2 min. Mass spectra were recorded at 70 eV and scanned in the range 40–400 m/z. Data were acquired and processed using Agilent GC/MS ChemStation software (B.07.04). The components were identified by comparing their mass spectra with the mass spectra stored in the NIST 2020 or reported in the literature. Identification was also performed by comparing their retention indices (RI) with the values reported in the literature [58]. The linear retention indices were determined in relation to the retention times (t_R_) of a homologous series of n-alkanes (C8–C24) analyzed under the same operating conditions. The relative amounts of the components, expressed as percentages, were calculated by a normalization procedure based on the peak area in the total ion chromatogram.

## 4. Conclusions

A fast and effective supercritical extraction of volatile terpenes from leaves and flowering tops of *A. annua* was optimized and compared with conventional hydrodistillation and steam distillation. Previous relevant research has mainly focused on artemisinin-rich extracts. To our knowledge, this is the first work to present the results of optimizing the sc-CO_2_ extraction of the other targeted monoterpenes and sesquiterpenes along with the extraction yield. The supercritical extracts contained a high proportion of monoterpenes (59.43–80.16%), with artemisia ketone, camphor and 1,8-cineole being the most abundant. The sesquiterpene fraction was dominated by arteannuin B and β-selinene. Compared to the conventional distillation methods used in this study, the sc-CO_2_ extracts contained significantly more sesquiterpenes, while the monoterpene fraction was reduced. The extraction yield (0.62–1.92%) was significantly affected by temperature, while different effects of pressure and temperature on the main constituents were observed. Our results demonstrate the optimal conditions for obtaining extracts with high contents of selected ingredients while maintaining high extraction yields. In contrast to conventional extraction techniques, this selective extraction of volatile terpenes could be useful to predict the biological effects of sc-CO_2_ extracts from *A. annua* and guide future research. In light of previous results, the high content of artemisia ketone and camphor in the optimized extracts would support the antimicrobial and antioxidant effects, while the high content of arteannuin B and β-selinene would contribute to the antiviral, anti-inflammatory and antitumor effects. The results obtained may contribute to the improving methods of extraction of bioactive plant ingredients for the pharmaceutical, food and cosmetic industries and support the development of high-quality natural products with minimal impact on the environment.

## Figures and Tables

**Table 1:** Composition of the essential oil of *Artemisia annua* L. obtained by hydrodistillation and steam distillation.

		Yield (%)	
Compound	RI	Hydrodistillation	Steam Distillation
Tricyclene	922	0.17	0.11
α-Thujene	926	-	0.20
α-Pinene	933	7.64	17.78
2-Methylpropyl butanoate	943	0.17	0.27
Camphene	947	3.86	2.55
Sabinene	973	1.51	1.25
β-Pinene	976	1.29	1.84
Myrcene	991	7.73	1.11
Yomogi alcohol	1001	0.93	0.39
α-Terpinene	1016	0.17	0.31
*p*-Cymene	1024	-	0.72
Limonene	1028	0.26	0.23
1,8-Cineole	1030	9.88	7.47
γ-Terpinene	1057	0.22	0.42
Artemisia ketone	1061	29.20	42.04
*cis*-Sabinene hydrate	1067	0.19	-
Artemisia alcohol	1083	3.22	0.81
*trans*-Sabinene hydrate	1099	0.19	-
1,3,8-*p*-Mentatriene	1103	1.59	-
α-Campholenal	1126	0.24	0.47
*trans*-Pinocarveol	1138	0.92	1.79
2-Methyl-6-methylene-1,7-octadien-3-one	1140	0.72	-
Camphor	1143	17.67	4.89
Ocimenol	1157	1.17	-
Pinocarvone	1162	1.20	2.46
Terpinen-4-ol	1177	0.54	0.29
α-Terpineol	1190	0.56	-
Myrtenol	1196	0.55	0.73
Eugenol	1357	0.22	-
α-Copaene	1374	0.37	0.33
*trans*-β-Caryophyllene	1417	0.92	-
*trans*-β-Farnesene	1456	0.31	-
Artemisia triene	1465	0.20	0.35
Germacrene D	1478	1.10	-
β-Selinene	1483	1.74	3.70
Caryophyllene oxide	1580	0.90	2.26
Monoterpenes		91.62	88.13
Hydrocarbons		24.82	26.79
Oxidized forms		66.80	61.34
Sesquiterpenes		5.54	6.64
Hydrocarbons		4.64	4.38
Oxidized forms		0.90	2.26
Others		0.39	0.27
TOTAL		97.55	95.04

RI—retention index on HP-5 MS column.

**Table 2:** The yield of sc-CO_2_ extracts of *Artemisia annua* L. as a function of pressure and temperature.

Sequence	Pressure (bar)	Temperature (°C)	Yield (%)
1	220	60	1.43
2	130	50	0.90
3	100	60	1.92
4	160	60	1.61
5	220	40	1.05
6	190	50	1.46
7	190	60	1.88
8	130	40	0.62
9	220	50	1.27
10	160	40	0.89
11	100	50	1.59
12	160	50	1.10
13	130	60	1.55
14	190	40	0.66
15	100	40	1.02

**Table 3:** Chemical composition of sc-CO_2_ extracts of *Artemisia annua* L.

Compound	RI	1	2	3	4	5	6	7	8	9	10	11	12	13	14	15
Santolina triene	908	-	-	-	-	-	-	-	-	0.22	-	-	-	-	-	-
α-Pinene	933	0.77	2.75	2.34	2.60	1.31	2.11	3.30	2.48	2.99	1.08	3.99	2.44	1.05	0.95	0.76
Camphene	947	-	0.39	0.52	0.40	0.26	-	-	-	0.51	0.39	-	-	-	-	-
Sabinene	973	-	0.39	0.33	0.39	0.27	-	0.57	0.53	0.50	0.35	0.70	0.53	0.34	-	-
β-Pinene	976	-	0.43	0.37	0.39	0.24	-	0.44	-	0.43	-	0.54	0.35	-	-	-
Myrcene	991	0.45	1.35	1.32	0.87	0.71	0.81	0.60	0.93	0.80	0.80	0.82	1.46	0.89	0.83	0.68
Yomogi alcohol	1001	0.50	0.27	-	-	0.42	-	-	-	0.34	0.56	-	0.43	0.49	0.52	0.68
1,8-Cineole	1031	6.64	8.05	8.00	8.24	6.79	10.35	11.29	11.24	7.06	7.44	11.89	7.62	8.59	6.94	4.77
Artemisia ketone	1061	31.55	19.28	19.57	16.93	27.06	38.04	19.90	30.13	27.43	48.49	25.31	31.75	29.81	37.56	46.76
*cis*-Sabinene hydrate	1066	1.24	1.84	1.80	1.91	1.31	1.41	2.30	2.04	1.01	0.68	2.35	1.49	1.43	0.90	0.94
Artemisia alcohol	1083	3.64	2.70	2.43	2.25	3.08	4.03	2.81	3.60	2.50	3.44	3.24	2.72	2.97	2.94	4.10
*trans*-Sabinene hydrate	1099	0.65	0.76	0.55	0.83	0.67	-	1.01	0.91	0.58	0.38	1.03	0.69	0.81	0.56	0.44
1,3,8-*p*-Mentatriene	1103	1.11	2.18	2.41	2.17	1.66	0.93	0.93	1.25	0.89	-	0.93	1.00	1.10	0.75	-
α-Campholenal	1126	0.78	1.22	1.31	1.37	0.82	0.78	1.24	0.90	0.78	0.37	1.15	0.75	0.71	-	-
*trans*-Pinocarveol	1138	2.66	2.96	2.80	3.52	3.01	2.59	5.08	3.68	2.74	1.39	4.81	2.90	2.54	1.42	1.74
Camphor	1143	13.94	14.17	18.44	13.15	11.88	16.05	3.29	12.88	9.39	12.22	4.17	7.17	6.77	14.36	7.83
Ocimenol	1157	1.09	1.99	2.32	2.01	1.37	1.02	1.13	1.20	0.97	-	1.08	1.02	1.28	0.81	-
Pinocarvone	1162	1.86	1.86	1.79	2.23	1.93	2.03	3.31	2.45	1.85	1.28	3.10	1.97	1.95	1.15	1.53
Borneole	1165	-	0.13	-	-	-	-	0.23	-	0.12	-	-	-	0.75	-	-
α-Terpineol	1190	0.60	0.77	0.55	0.68	0.51	-	0.88	0.98	0.59	0.36	0.98	0.83	0.85	-	0.54
Myrtenol	1196	0.70	0.77	0.66	0.88	0.70	-	1.09	0.86	0.71	0.41	1.06	0.80	0.72	-	-
Eugenol	1357	-	-	-	-	-	-	-	-	0.48	-	-	-	-	-	-
α-Copaene	1374	1.76	1.62	1.23	1.50	2.32	1.76	1.82	1.78	1.24	1.33	1.82	1.98	1.64	1.70	1.90
*trans*-β-Caryophyllene	1417	3.15	2.87	2.68	3.23	5.41	3.95	4.04	3.37	2.59	2.53	3.86	3.75	3.33	4.19	3.78
*trans*-β-Farnesene	1457	0.96	0.98	0.77	0.89	1.53	1.23	0.88	0.86	0.91	0.52	0.75	1.32	1.03	1.29	1.31
Artemisia triene	1465	0.50	0.68	0.48	0.78	0.56	-	0.68	0.60	0.75	0.53	0.58	0.98	-	0.78	1.06
Germacrene D	1478	1.71	1.71	1.66	1.85	3.79	3.48	2.54	2.75	1.74	1.98	2.56	3.42	3.38	5.33	4.34
β-Selinene	1483	1.87	5.26	5.16	6.41	4.98	1.05	7.42	3.80	4.91	3.60	7.14	6.72	6.23	4.26	5.88
Caryophyllene oxide	1580	1.82	2.00	1.36	1.83	2.10	1.55	2.88	-	2.22	1.14	2.43	2.08	1.53	1.65	1.40
Eudesma-4(15),7-dien-1-β-ol	1683	-	0.58	-	0.57	0.55	-	-	-	-	-	-	0.92	1.02	-	-
Arteannuic acid	1840	0.80	0.55	1.55	0.62	0.58	-	0.61	1.72	0.55	-	0.74	0.20	0.52	-	-
Arteannuin B	2054	10.03	6.10	5.82	7.55	6.23	4.23	8.86	3.98	9.52	4.38	6.70	7.05	7.54	8.32	5.86
Monoterpenes		68.18	62.08	67.51	60.80	63.98	80.16	59.43	76.06	62.41	79.66	67.13	65.90	63.06	69.68	70.75
Hydrocarbons		4.23	7.91	9.64	9.56	6.44	5.26	9.17	8.13	7.92	3.68	10.34	7.95	5.63	3.99	2.82
Oxidized forms		63,95	54,17	57,87	51,24	57,54	74,90	50,26	67,93	54,49	75,98	56,79	57,95	57,43	65,69	67,93
Sesquiterpenes		22.59	22.34	20.69	25.24	28.05	17.24	29.73	18.85	24.43	15.99	26.57	28.41	26.22	27.51	25.52
Hydrocarbons		9.95	13.11	11.96	14.66	18.59	11.46	17.38	13.16	12.14	10.47	16.70	18.16	15.61	17.54	18.26
Oxidized forms		1.64	9.23	8.73	10.58	9.46	5.78	12,35	5.71	12.29	5.52	9.87	10.25	10.61	9.97	7.26
Phenylpropanes		-	-	-	-	-	-	-	-	0.48	-	-	-	-	-	-
TOTAL		90.77	84.43	88.20	86.04	92.03	97.39	89.16	94.91	87.32	95.64	93.70	94.30	89.27	97.19	96.27

RI—retention index on HP-5 MS column; 1–15: number of sequences corresponding to the sc-CO_2_ extracts listed in Table 2.

**Table 4:** Optimal pressure and temperature values for targeted sc-CO_2_ extraction of volatile terpenes from *Artemisia annua* L.

	Target	Pressure (bar)	Temperature (°C)	Desirability
1.	High extraction yield and high monoterpenes	100	60	0.660
2.	High extraction yield and high artemisia ketone	100	45	0.473
3.	High extraction yield and high artemisia ketone, camphor and 1,8-cineole	160	50	0.509
4.	High extraction yield and high sesquiterpenes	181	60	0.809
5.	High extraction yield and high arteannuin B	220	60	0.899
6.	High extraction yield and high arteannuin B and β-selinene	220	53	0.650

## Data Availability

Data are contained within the article and Supplementary Materials.

## Supplementary Materials

The following supporting information can be downloaded at https://www.mdpi.com/article/10.3390/molecules30071638/s1, Table S1: Statistical analysis of response surface quadratic models for extraction yield; Table S2: Statistical analysis of response surface quadratic models for total monoterpenes; Table S3: Statistical analysis of response surface quadratic models for total sesquiterpenes; Table S4: Statistical analysis of response surface quadratic models for artemisia ketone; Table S5: Statistical analysis of response surface quadratic models for 1,8-cineole; Table S6: Statistical analysis of response surface quadratic models for camphor; Table S7: Statistical analysis of response surface quadratic models for arteannuin B; Table S8: Statistical analysis of response surface quadratic models for β-selinene.

## References

[1] Sanz M., Vilatersana R., Hidalgo O., Garcia-Jacas N., Susanna A., Schneeweiss G.M., Vallès J. (2008). Molecular phylogeny and evolution of floral characters of *Artemisia* and allies (Anthemideae, Asteraceae): Evidence from NrDNA ETS and ITS sequences. Taxon.

[2] Anibogwu R., Jesus K.D., Pradhan S., Pashikanti S., Mateen S., Sharma K. (2021). Extraction, isolation and characterization of bioactive compounds from *Artemisia* and their biological significance: A review. Molecules.

[3] Bisht D., Kumar D., Kumar D., Dua K., Kumar Chellappan D. (2021). Phytochemistry and pharmacological activity of the genus *Artemisia*. Arch. Pharm. Res..

[4] Nigam M., Atanassova M., Mishra A.P., Pezzani R., Prasad Devkota H.P., Plygun S., Salehi B., Setzer W.N., Sharifi-Rad J. (2019). Bioactive compounds and health benefits of *Artemisia* species. Nat. Prod. Commun..

[5] Hussain M., Thakurn R.K., Khazir J., Ahmed S., Khan M.I., Rahi P., Peer L.A., Shanmugam P.V., Kaur S., Raina S.N. (2024). Traditional uses, phytochemistry, pharmacology, and toxicology of the genus *Artemisia* L. (Asteraceae): A high-value medicinal plant. Curr. Top. Med. Chem..

[6] Su X.-Z., Miller L.H. (2015). The discovery of artemisinin and Nobel Prize in Physiology or Medicine. Sci. China Life Sci..

[7] Ma N., Zhang Z., Liao F., Jiang T., Tu Y. (2020). The birth of artemisinin. Pharmacol. Ther..

[8] Ekiert H., Świątkowska J., Klin P., Rzepiela A., Szopa A. (2021). *Artemisia annua*—Importance in traditional medicine and current state of knowledge on the chemistry, biological activity and possible applications. Planta Med..

[9] Efferth T. (2017). From ancient herb to modern drug: *Artemisia annua* and artemisinin for cancer therapy. Semin. Cancer Biol..

[10] Feng X., Cao S., Qui F., Zang B. (2020). Traditional application and modern pharmacological research of *Artemisia annua* L.. Pharmacol. Ther..

[11] Septembre-Malaterre A., Lalarizo Rakoto M., Marodon C., Bedoui Y., Nakab J., Simon E., Hoarau L., Savriama S., Strasberg D., Guiraud P. (2020). *Artemisia annua*, a traditional plant brought to light. Int. J. Mol. Sci..

[12] Baggieri M., Gioacchini S., Borgonovo G., Catinella G., Marchi A., Picone P., Vasto S., Fioravanti R., Bucci P., Kojouri M. (2023). Antiviral, virucidal and antioxidant properties of *Artemisia annua* against SARS-CoV-2. Biomed. Pharmacother..

[13] Ekiert H., Klimek-Szczykutowicz M., Rzepiela A., Klin P., Szopa A. (2022). *Artemisia* species with high biological values as a potential source of medicinal and cosmetic raw materials. Molecules.

[14] Sharopov F.S., Salimov A., Numonov S., Safomuddin A., Bakri M., Salimov T., Setzer W.N., Habasi M. (2020). Chemical composition, antioxidant, and antimicrobial activities of the essential oils from *Artemisia annua* L. growing wild in Tajikistan. Nat. Prod. Commun..

[15] Bilia A.R., Santomauro F., Sacco C., Bergonzi M.C., Donato R. (2014). Essential oil of *Artemisia annua* L.: An extraordinary component with numerous antimicrobial properties. Evid. Based Complement. Alternat. Med..

[16] Hong M., Kim M., Jang H., Bo S., Deepa P., Sowndhararajan K., Kim S. (2023). Multivariate analysis of essential oil composition of *Artemisia annua* L. collected from different locations in Korea. Molecules.

[17] Li Y., Xia L., Vazquez J.F.T., Song S. (2017). Optimization of supercritical CO_2_ extraction of essential oil from *Artemisia annua* L. by means of response surface methodology. J. Essent. Oil Bear. Plants.

[18] Rao A.B., Sardeshpande V.R. (2023). A hydrodistillation-based essential oils extraction: A quest for the most effective and cleaner technology. Sustain. Chem. Pharm..

[19] Yousefi M., Rahimi-Nasrabadi M., Pourmortazavi S.M., Wysokowski M., Jesionowski T., Ehrlich H., Mirsadeghi S. (2019). Supercritical fluid extraction of essential oils. TrAC Anal. Chem..

[20] Cheriyan B.V., Karunakar K.K., Anandakumar R., Murugathirumal A., Kumar A.S. (2025). Eco-friendly extraction technologies: A comprehensive review of modern green analytical methods. Sustain. Chem. Clim. Action..

[21] Zhao S., Zhang D. (2014). Supercritical CO_2_ extraction of *Eucalyptus* leaves oil and comparison with Soxhlet extraction and hydro-distillation methods. Sep. Purif. Tecnol..

[22] Šulniūtė V., Baranauskienė R., Ragažinskien O., Rimantas Venskutonis P. (2017). Comparison of composition of volatile compounds in ten *Salvia* species isolated by different methods. Flavor. Fragr. J..

[23] Bendif H., Adouni K., Miara M.D., Baranauskienė R., Kraujalis P., Venskutonis P.R., Nabavi S.M., Maggi F. (2018). Essential oils (EOs),pressurized liquid extracts (PLE) and carbon dioxide supercritical fluid extracts (SFE-CO_2_) from Algerian *Thymus munbyanus* as valuable sources of antioxidants to be used on an industrial level. Food Chem..

[24] Donato R., Santomauro F., Bilia A.R., Flamini G., Sacco C. (2015). Antibacterial activity of Tuscan *Artemisia annua* essential oil and its major components against some foodborne pathogens. LWT-Food Sci. Technol..

[25] Radulović N.S., Randjelović P.J., Stojanović N.M., Blagojević P.D., Stojanović-Radić Z.Z., Ilić I.R., Djordjević V.B. (2013). Toxic essential oils. Part II: Chemical, toxicological, pharmacological and microbiological profiles of *Artemisia annua* L. volatiles. Food Chem. Toxicol..

[26] Grifoni L., Sacco C., Donato R., Tziakas S., Tomou E.-M., Skaltsa H., Vanti G., Bergonzi M.C., Bilia A.R. (2024). Environmentally friendly microemulsions of essential oils of *Artemisia annua* and *Salvia fruticosa* to protect crops against *Fusarium verticillioides*. Nanomaterials.

[27] Chebbac K., Benziane Ouaritini Z., El Moussaoui A., Chalkha M., Lafraxo S., Bin Jardan Y.A., Nafidi H.-A., Bourhia M., Guemmouh R. (2023). Antimicrobial and antioxidant properties of chemically analyzed essential oil of *Artemisia annua* L. (Asteraceae) native to Mediterranean area. Life.

[28] Marinas I.C., Oprea E., Chifiriuc M.C., Badea I.A., Buleandra M., Lazar V. (2015). Chemical composition and antipathogenic activity of *Artemisia annua* essential oil from Romania. Chem. Biodivers..

[29] Risaliti L., Pini G., Ascrizzi R., Donato R., Sacco C., Bergonzi M.C., Salvatici M.C., Bilia A.R. (2020). *Artemisia annua* essential oil extraction, characterization, and incorporation in nanoliposomes, smart drug delivery systems against *Candida* species. J. Drug Deliv. Sci. Technol..

[30] Vidic D., Čopra-Janićijević A., Miloš M., Maksimović M. (2018). Effects of different methods of isolation on volatile composition of *Artemisia annua* L.. Int. J. Anal. Chem..

[31] Bedinim S., Flamini G., Cosci F., Ascrizzi R., Echeverria M.C., Guidi L., Landi M., Lucchi A., Conti B. (2017). *Artemisia* spp. essential oils against the disease-carrying blowfly *Calliphora vomitoria*. Parasites Vectors.

[32] Zhigzhitzhapova S.V., Dylenova E.P., Gulyaev S.M., Randalova T.E., Taraskin V.V., Tykheev Z.A., Radnaeva L.D. (2020). Composition and antioxidant activity of the essential oil of *Artemisia annua* L.. Nat. Prod. Res..

[33] Santomauro F., Donato R., Sacco C., Pini G., Flamini G., Bilia A.F. (2016). Vapour and liquid-phase *Artemisia annua* essential oil activities against several clinical strains of *Candida*. Planta Med..

[34] Aćimović M., Stanković Jeremić J., Todosijević M., Kiprovski B., Vidović S., Vladić J., Pezo L. (2022). Comparative study of the essential oil and hydrosol composition of sweet wormwood (*Artemisia annua* L.) from Serbia. Chem. Biodivers..

[35] Ćavar S., Vidic D., Parić A. (2012). Chemical composition and antioxidant and antimicrobial activity of essential oil of *Artemisia annua* L. from Bosnia. Ind. Crop Prod..

[36] Liu H., Guo S.-S., Lu L., Li D., Liang J., Huang Z.-H., Zhou Y.-M., Zhang W.-J., Du S. (2021). Essential oil from *Artemisia annua* aerial parts: Composition and repellent activity against two storage pests. Nat. Prod. Res..

[37] Oftadeh M., Sendi J.J., Ebadollahi M. (2020). Toxicity and deleterious effects of *Artemisia annua* essential oil extracts on mulberry pyralid (*Glyphodes pyloalis*). Pestic. Biochem. Physiol..

[38] Perazzo F.F., Carvalho J.C.T., Rehder V.L.G. (2003). Central properties of the essential oil and the crude ethanol extract from aerial parts of *Artemisia annua* L.. Pharmacol. Res..

[39] Habibi Z., Ghanian S., Ghasemi S., Yousefi M. (2013). Chemical composition and antibacterial activity of the volatile oil from seeds of *Artemisia annua* L. from Iran. Nat. Prod. Res..

[40] Ma L., Wei L., Chen X., Wang W., Lu J., Li Y., Yao L. (2024). Chemical composition, antioxidative and antimicrobial activities of essential oil of wild *Artemisia annua* from Ningxia, China. Nat. Prod. Res..

[41] Das S., Vörös-Horváth B., Bencsik T., Micalizzi G., Mondello L., Horváth G., Kőszegi T., Széchenyi A. (2020). Antimicrobial activity of different *Artemisia* essential oil formulations. Molecules.

[42] Juteau F., Masotti V., Bessière J.M., Dherbomez M., Viano J. (2002). Antibacterial and antioxidant activities of *Artemisia annua* essential oil. Fitoterapia.

[43] Kohler M., Haerdi W., Christen P., Veuthey J.L. (1997). Extraction of artemisinin and artemisinic acid from *Artemisia annua* L. using supercritical carbon dioxide. J. Chromatogr. A.

[44] Baldino L., Reverchon E., Della Porta G. (2017). An optimized process for SC-CO_2_ extraction of antimalarial compounds from *Artemisia annua* L.. J. Supercrit. Fluids.

[45] Martinez-Correa H.A., Bitencourt R.G., Kayano A.C.A.V., Magalhães P.M., Costa F.T.M., Cabral F.A. (2017). Integrated extraction process to obtain bioactive extracts of *Artemisia annua* L. leaves using supercritical CO_2_, ethanol and water. Ind. Crops Prod..

[46] Ciftci O.N., Cahyadi J., Guigard S.E., Saldaña M.D.A. (2018). Optimization of artemisinin extraction from *Artemisia annua* L. with supercritical carbon dioxide + ethanol using response surface methodology. Electrophoresis.

[47] Vidović S., Simić S., Gavarić A., Aćimović M., Vladic J. (2020). Extraction of sweet wormwood (*Artemisia annua* L.) by supercritical carbon dioxide. Lek. Sirovine.

[48] Confortin T.C., Todero I., Canabarro N., Luft L., Ugalde G.A., Neto J.R.C., Mazutti M.A., Zabot G.L., Tres M.V. (2019). Supercritical CO_2_ extraction of compounds from different aerial parts of *Senecio brasiliensis*: Mathematical modeling and effects of parameters on extract quality. J. Supercrit. Fluids.

[49] Vladić J., Jerković I., Svilović S., Pavić V., Pastor K., Paiva A., Jokić S., Rebocho S., Duarte A.R. (2023). Evaluation of the volatiles’ chemical profile and antibacterial activity of *Lavandula stoechas* L. extracts obtained by supercritical carbon dioxide. Sustain. Chem. Pharm..

[50] Banožić M., Wronska A.W., Jakovljević Kovač M., Aladić K., Jerković I., Jokić S. (2023). Comparative evaluation of different extraction techniques for separation of artemisinin from sweet wormwood *(Artemisia annua* L.). Horticulturae.

[51] Wang Y., Huang W., Wang N., Ouyang D., Xiao L., Zhang S., Ou X., He T., Yu R., Song L. (2021). Development of arteannuin B sustained-release microspheres for anti-tumor therapy by integrated experimental and molecular modeling approaches. Pharmaceutics.

[52] Huang W., Wang Y., He T., Zhu J., Li J., Zhang S., Zhu Y., Xu Y., Xu L., Wang H. (2022). Arteannuin B enhances the effectiveness of cisplatin in non-small cell lung cancer by regulating connexin 43 and MAPK pathway. Am. J. Chin. Med..

[53] Varela K., Arman H.D., Berger M.S., Sponsel V.M., Lin C.A., Yoshimoto F.K. (2023). Inhibition of cysteine proteases via thiol-Michael addition explains the anti-SARS-CoV-2 and bioactive properties of arteannuin B. J. Nat. Prod..

[54] Chen H., Hu Q., Wen T., Luo L., Liu L., Wang L., Shen X. (2024). Arteannuin B, a sesquiterpene lactone from *Artemisia annua*, attenuates inflammatory response by inhibiting the ubiquitin-conjugating enzyme UBE2D3-mediated NF-kappaB activation. Phytomedicine.

[55] Chandra M., Prakash O., Kumar R., Bachheti R.K., Bhushan B., Kumar M., Pant A.K. (2017). *β*-Selinene-rich essential oils from the parts of *Callicarpa macrophylla* and their antioxidant and pharmacological activities. Medicines.

[56] Maiolini T.C.S., Nicácio K.J., Rosa W., Miranda D.O., Santos M.F.C., Bueno P.C.P., Lago J.H.G., Sartorelli P., Dias D.F., de Chagas Paula D.A. (2025). Potential anti-inflammatory biomarkers from Myrtaceae essential oils revealed by untargeted metabolomics. Nat. Prod. Res..

[57] European Pharmacopoeia Online (11.6). https://pheur.edqm.eu/home.

[58] Adams R. (2007). Identification of Essential Oil Components by Gas Chromatography/Mass Spectrometry.

