# Supplementary material for: Green Extraction of Volatile Terpenes from Artemisia annua L
Source: Molecules. 2025 Apr 7;30(7):1638. doi: 10.3390/molecules30071638 (PMC11990131; doi:10.3390/molecules30071638)
Supplement: Supplementary file 1 [file molecules-30-01638-s001.zip › molecules-3513814-supplementary.pdf]

Supplementary materials for

Green Extraction of Volatile Terpenes from *Artemisia annua* L.

Marta Mandić [1], Ivona Ivančić [1], Matija Cvetnić [2], Claudio Ferrante [3], Giustino Orlando [3] and Sanda Vladimir-Knežević [4,*]

[1] Faculty of Pharmacy, University of Mostar, Matice Hrvatske BB, 88000 Mostar, Bosnia and Herzegovina; marta.mandic@farf.sum.ba (M.M.); ivona.ivancic@farf.sum.ba (I.I.)
[2] Faculty of Chemical Engineering and Technology, University of Zagreb, Trg Marka Marulića 19, 10000 Zagreb, Croatia; mcvetnic@fkit.unizg.hr
[3] Department of Pharmacy, D'Annunzio University of Chieti-Pescara, Via dei Vestini, 31, 66013 Chieti, Italy; claudio.ferrante@unich.it (C.F.); giustino.orlando@unich.it (G.O.)
[4] Faculty of Pharmacy and Biochemistry, University of Zagreb, Ante Kovačića 1, 10000 Zagreb, Croatia
* Correspondence: sanda.vladimir@pharma.unizg.hr

Table S1. Statistical analysis of response surface quadratic models for extraction yield.

Analysis of variance (ANOVA)					
Source	Sum of squares	Degree of freedom	Mean square	*F*-value	*p*-value
Model	1.80	5	0.36	5.65	0.0125
A	0.01	1	0.01	0.21	0.6591
B	1.59	1	1.59	25.03	0.0007
AB	0.06	1	0.06	0.90	0.3675
A^2	0.13	1	0.13	1.96	0.1947
B^2	0.01	1	0.01	0.13	0.7310
Residual	0.57	9	0.06		
Corr total	2.37	14			

Statistical parameters of the model							
Standard deviation	Mean	C.V. (%)	PRESS	R^2	Adjusted R^2	Predicted R^2	Adequate Precision
0.25	1.26	19.96	1.84	0.76	0.62	0.22	7.30

A - pressure; B - temperature; C.V. - coefficient of variation; Corr total - corrected total sum of squares; PRESS - predicted residual sum of squares for the model; R^2 – coefficient of determination; $p < 0.05$ is considered significant

Table S2. Statistical analysis of response surface quadratic models for total monoterpenes.

Analysis of variance (ANOVA)					
Source	Sum of squares	Degree of freedom	Mean square	*F*-value	*p*-value
Model	375.58	8	46.95	4.25	0.0473
A	0.73	1	0.73	0.07	0.8054
B	305.03	1	305.03	27.62	0.0019
AB	15.56	1	15.56	1.41	0.2801
A^2	4.18	1	4.18	0.38	0.5608
B^2	18.80	1	18.80	1.70	0.2397
A^2B	141.19	1	141.19	12.79	0.0117
AB^2	6.32	1	6.32	0.57	0.4778
A^3	1.60	1	1.60	0.15	0.7162
B^3	0	0			
Residual	66.25	6	11.04		
Corr total	441.83	14			

Statistical parameters of the model							
Standard deviation	Mean	C.V. (%)	PRESS	R^2	Adjusted R^2	Predicted R^2	Adequate Precision
3.23	67.12	4.95	530.79	0.85	0.65	-0.20	6.69

A - pressure; B - temperature; C.V. - coefficient of variation; Corr total - corrected total sum of squares; PRESS - predicted residual sum of squares for the model; R^2 – coefficient of determination; $p < 0.05$ is considered significant

Table S3. Statistical analysis of response surface quadratic models for total sesquiterpenes.

Analysis of variance (ANOVA)					
Source	Sum of squares	Degree of freedom	Mean square	*F*-value	*p*-value
Model	147.02	8	18.38	4.42	0.0433
A	4.95	1	4.95	1.19	0.3169
B	96.63	1	96.63	23.25	0.0029
AB	0.58	1	0.58	0.14	0.7215
A^2	9.08	1	9.08	2.19	0.1898
B^2	0.70	1	0.70	0.17	0.6951
A^2B	95.25	1	95.25	22.92	0.0030
AB^2	10.25	1	10.25	2.47	0.1674
A^3	10.64	1	10.64	2.56	0.1608
B^3	0	0			
Residual	24.93	6	4.16		
Corr total	171.95	14			

Statistical parameters of the model							
Standard deviation	Mean	C.V. (%)	PRESS	R^2	Adjusted R^2	Predicted R^2	Adequate Precision
2.04	23.89	8.53	147.40	0.85	0.66	0.14	6.83

A - pressure; B - temperature; C.V. - coefficient of variation; Corr total - corrected total sum of squares; PRESS - predicted residual sum of squares for the model; R^2 – coefficient of determination; $p < 0.05$ is considered significant

Table S4. Statistical analysis of response surface quadratic models for artemisia ketone.

Analysis of variance (ANOVA)					
Source	Sum of squares	Degree of freedom	Mean square	*F*-value	*p*-value
Model	932.62	5	186.52	4.25	0.0291
A	7.42	1	7.42	0.17	0.6904
B	838.80	1	838.80	19.12	0.0018
AB	62.52	1	62.52	1.43	0.2631
A^2	4.48	1	4.48	0.10	0.7567
B^2	19.40	1	19.40	0.44	0.5228
Residual	394.82	9	43.87		
Corr total	1327.44	14			

Statistical parameters of the model							
Standard deviation	Mean	C.V. (%)	PRESS	R^2	Adjusted R^2	Predicted R^2	Adequate Precision
6.62	27.97	23.68	1149.30	0.70	0.54	0.13	6.06

A - pressure; B - temperature; C.V. - coefficient of variation; Corr total - corrected total sum of squares; PRESS - predicted residual sum of squares for the model; R^2 – coefficient of determination; $p < 0.05$ is considered significant

Table S5. Statistical analysis of response surface quadratic models for 1,8-cineole.

Analysis of variance (ANOVA)							
Source	Sum of squares	Degree of freedom	Mean square	*F*-value	*p*-value		
Model	34.68	5	6.94	3.54	0.0480		
A	2.51	1	2.51	1.28	0.2877		
B	4.72	1	4.72	2.41	0.1553		
AB	4.10	1	4.10	2.09	0.1823		
A^2	0.78	1	0.78	0.40	0.5428		
B^2	22.57	1	22.57	11.51	0.0080		
Residual	17.66	9	1.96				
Corr total	52.34	14					
Statistical parameters of the model							
Standard deviation	Mean	C.V. (%)	PRESS	R^2	Adjusted R^2	Predicted R^2	Adequate Precision
1.40	7.86	17.82	54.70	0.66	0.48	-0.04	4.87

A - pressure; B - temperature; C.V. - coefficient of variation; Corr total - corrected total sum of squares; PRESS - predicted residual sum of squares for the model; R^2 – coefficient of determination; $p < 0.05$ is considered significant

Table S6. Statistical analysis of response surface quadratic models for camphor.

Analysis of variance (ANOVA)					
Source	Sum of squares	Degree of freedom	Mean square	*F*-value	*p*-value
Model	148.98	5	29.80	5.99	0.0104
A	44.08	1	44.08	8.86	0.0155
B	0.12	1	0.12	0.02	0.8785
AB	39.75	1	39.75	7.99	0.0198
A^2	64.93	1	64.93	13.06	0.0056
B^2	0.09	1	0.09	0.017	0.8985
Residual	44.76	9	4.97		
Corr total	193.73	14			

Statistical parameters of the model							
Standard deviation	Mean	C.V. (%)	PRESS	R^2	Adjusted R^2	Predicted R^2	Adequate Precision
2.23	10.08	22.12	120.08	0.77	0.64	0.38	8.22

A - pressure; B - temperature; C.V. - coefficient of variation; Corr total - corrected total sum of squares; PRESS - predicted residual sum of squares for the model; R^2 – coefficient of determination; $p < 0.05$ is considered significant

Table S7. Statistical analysis of response surface quadratic models for arteannuin B.

Analysis of variance (ANOVA)					
Source	Sum of squares	Degree of freedom	Mean square	*F*-value	*p*-value
Model	30.91	5	6.18	4.57	0.0237
A	12.76	1	12.76	9.43	0.0133
B	3.51	1	3.51	2.60	0.1416
AB	9.52	1	9.52	7.03	0.0264
A^2	4.88	1	4.88	3.61	0.0901
B^2	0.24	1	0.24	0.18	0.6855
Residual	12.18	9	1.35		
Corr total	43.09	14			

Statistical parameters of the model							
Standard deviation	Mean	C.V. (%)	PRESS	R^2	Adjusted R^2	Predicted R^2	Adequate Precision
1.16	6.74	17.25	34.91	0.72	0.56	0.19	7.29

A - pressure; B - temperature; C.V. - coefficient of variation; Corr total - corrected total sum of squares; PRESS - predicted residual sum of squares for the model; R^2 – coefficient of determination; $p < 0.05$ is considered significant

Table S8. Statistical analysis of response surface quadratic models for *β*-selinene.

Analysis of variance (ANOVA)					
Source	Sum of squares	Degree of freedom	Mean square	*F*-value	*p*-value
Model	18.20	5	3.64	4.48	0.0251
A	8.12	1	8.12	9.99	0.0115
B	3.62	1	3.62	4.46	0.0640
AB	0.43	1	0.43	0.52	0.4874
A^2	0.17	1	0.17	0.20	0.6617
B^2	5.87	1	5.87	7.22	0.0249
Residual	7.31	9	0.81		
Corr total	25.51	14			

Statistical parameters of the model							
Standard deviation	Mean	C.V. (%)	PRESS	R^2	Adjusted R^2	Predicted R^2	Adequate Precision
0.90	4.83	18.67	19.36	0.71	0.55	0.24	7.54

A - pressure; B - temperature; C.V. - coefficient of variation; Corr total - corrected total sum of squares; PRESS - predicted residual sum of squares for the model; R^2 – coefficient of determination; $p < 0.05$ is considered significant
